# Understanding age-related modifications of motor control strategies

**DOI:** 10.1186/1743-0003-5-26

**Published:** 2008-11-11

**Authors:** Silvestro Micera

**Affiliations:** 1 Advanced Robotics Technology and Systems Lab, Scuola Superiore Sant'Anna, Pisa, Italy; 2 Institute for Automation, Swiss Federal Institute of Technology, Zurich, Switzerland

## Background

The last century handed us over a considerably older population, particularly in the developed countries. Life expectancy, which in the industrialized countries at the beginning of the 1900's, was barely 47 years, has progressively increased and today is almost 80 years, with women in an advantageous position. Moreover, the real qualitative leap is represented by the conditions in which ageing takes place, conditions that till now were inconceivable for past generations, such as the level of education, the health status and the economic resources.

**Figure 1:**
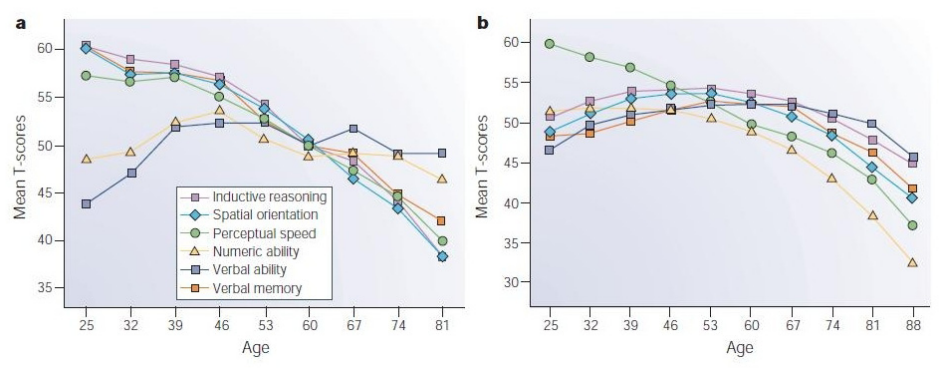
**Cross-sectional and longitudinal estimates of age-related change in cognition.** a) Cross-sectional data from the Seattle Longitudinal Study. B) Seven year longitudinal data from the same study [9].

Senior citizens are eager to maintain their quality of life but at the same time ageing poses them significant problems. This is due to the very important age-related modifications to the nervous and musculoskeletal systems. In fact, ageing modifies the properties of the neuromuscular system [1], reduces the conduction velocity of neural signals [2], and in general the performance in complex sensory-motor tasks increasing the reaction time to external stimuli [3]. Ageing is associated with a decrease in mental and cognitive skills and also with an important reduction in motor abilities, which are often cause of increased risk of accidents [4,5]. Specifically, elderly subjects are generally slower and less reactive than younger adults [6], and show higher muscle co-activation, reduced muscular force and power and reduced ability in force regulation [7]. Moreover, elders have difficulties with sensory discrimination, perceptual encoding, response selection and motion preparation [8]. Even in able-bodied elderly people, it is possible to observe a natural reduction of cognitive performance as shown in [9] (Figure 1).

The ability to maintain balance during locomotion is also naturally reduced due to ageing and this leads to fatigue and increased fall risks as the most important consequences [10]. Falling represents one of the most worrying events for elderly people because it involves several aspects of the autonomous daily life, healthy, costs for the single subjects and costs for the society in order to increase suitable structures and manage employees, therapists and assistance.

Differences can also be found in the analysis of reaching movements [11]. Interestingly, elders seem to deal with the modifications in their abilities by finding alternative solutions "customized" to their conditions and possibilities. This is something we could expect: the change in the properties of the "plant" asks for a redefinition of the control strategies to (try to) achieve the same level of performance.

**Figure 2:**
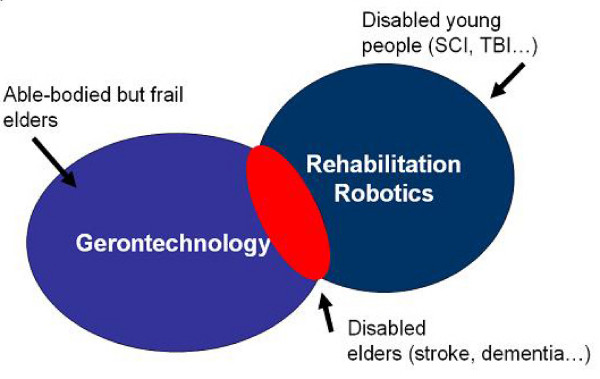
The relationship between "gerontechnology" and "rehabilitation robotics": the former addresses the development of systems which are not necessarily developed for disabled elders.

The analysis of the age-related modifications of motor control strategies is a topic of extreme interest for many social and clinical reasons because of the significant reduction of elders quality of life due to these issues. In fact, understanding these modifications is important not only to deep our basic scientific knowledge but also to develop more effective systems to improve the quality of life of elders. Many groups are now developing advanced technological solutions not only for seniors affected by disabilities but also for able-bodied (but frail) elders [12] (Figure 2). This new field of research (often called "gerontechnology" which is the combination of "gerontology" – the scientific study of aging – and "technology") cannot be successful without a thorough analysis of the age-related modifications of motor control strategies.

## Special issue on "Age-related modifications of motor control strategies"

The aim of this special issue (SI) was to show the results achieved by some interesting and promising activities in this specific field. Even if it was not possible to cover all the possible research subjects, many important aspects have been addressed by the authors of the manuscripts published in the SI.

Fradet and colleagues and Cesqui and colleagues studied how ageing affects the generation and control of upper limb movements with a particular attention to the generation of sub-movements and the learning of internal models to deal with modified dynamic environments, respectively.

Mazzà and colleagues investigated different aspects related to the coordination between upper and lower extremities during walking.

Bock and Priest and colleagues analyzed how increased cognitive efforts are able to modify the performance of elders and the motor control strategy they implement. This is a very important issue because elders are asked to deal with multiple challenging tasks in many activities of daily living

## Competing interests

The author declares that he has no competing interests.

## References

[B1] Hamerman D, Hazzard WR, Andres R, Bierman EL, Blass JP (1990). Aging and the musculoskeletal system. Principle of geriatric medicine and geronthology.

[B2] Mortimer JA, Pirozzolo FJ, Maletta JG (1982). The Aging Motor System.

[B3] Harridge SDR, Saltin B (1996). Neuromuscular System. Encyclopedia of gerontology.

[B4] Seidler RD, Stelmach GE (1996). Motor Control. Encyclopedia of gerontology.

[B5] Seidler RD, Stelmach GE (1998). Persistence in visual feedback control by the elderly. Exp Brain Res.

[B6] Darling WG, Cooke JD, Brown SH (1989). Control of simple arm movements in elderly humans. Neurobiol Aging.

[B7] Ketcham CJ, Dounskaia NV, Stelmach GE (2004). Age-related differences in the control of multijoint movements. Motor Control.

[B8] Bock O, Girgenrath M (2006). Relationship between sensorimotor adaptation and cognitive functions in younger and older subjects. Exp Brain Res.

[B9] Hedden T, Gabrieli JDE (2004). Insights into the ageing mind: a view from cognitive neuroscience. Nat Rev Neurosci.

[B10] Masud T, Morris RO (2001). Epidemiology of falls. Age and Ageing.

[B11] Lee G, Fradet L, Ketcham CJ, Dounskaia N (2007). Efficient control of arm movements in advanced age. Exp Brain Res.

[B12] Micera S, Bonato P, Tamura T (2008). Gerontechnology: Advanced solutions for an ageing sosciety.

